# Neuromechanism, recovery effect and case study of swimming training intervention in children with cerebral palsy: A case report

**DOI:** 10.1097/MD.0000000000035223

**Published:** 2023-12-15

**Authors:** Jing Zeng, Shuang Hao, Yuxuan Wang, Qing Liu

**Affiliations:** a Chengdu Sport University, Chengdu, Sichuan, China; b Postdoctoral Research Station of Physical Education, Shanxi University, Taiyuan, Shanxi, China; c Chengdu University of Traditional Chinese Medicine, Chengdu, Sichuan, China.

**Keywords:** balance ability, cerebral palsy-case report, dystonia, gait recovery, swimming training

## Abstract

**Background:**

Cerebral palsy, hereinafter referred to as “cerebral palsy”, refers to a non progressive injury that occurs during the development of brain tissue in fetuses or infants. The patients often have walking dysfunction, abnormal balance ability and abnormal body stability, which are mainly caused by Cranial nerves injury.

**Patient concerns:**

One child diagnosed with ataxia cerebral palsy by the hospital was recruited, aged 6 years and 9 months. The symptoms were: lower limb adduction and internal rotation, left neck tilt to the left due to insufficient muscle tension, and eyes squint to the right. The movement is clumsy and the coordination ability of limbs is poor; Its body balance function is poor, the sitting and standing position cannot keep the body upright and balanced for a long time, and the coordination of the random movement of hands and eyes is poor; Weak spatial cognition and orientation ability; Have persistent central motor dysfunction. When walking, the body leans forward and sideways, and the gait is staggered, which is easy to fall; In terms of expression, it shows vague language and unclear speech; Relatively retarded in intelligence.

**Diagnosis and intervention:**

The study used swimming training intervention to report a twelve months training intervention program for a child with ataxic cerebral palsy, and evaluated it with Berg balance scale and modified Ashworth scale.

**Outcomes:**

Swimming training has a significant effect on the rehabilitation of children with ataxic cerebral palsy; The forces from different directions in the water can improve the balance of children with cerebral palsy; Muscle endurance training with medium load intensity can help restore unilateral muscle tension deficiency to a certain extent, and make bilateral muscle tension gradually becomes.

consistent, thus enhancing the balance ability, gait and body stability of children with cerebral palsy.

## 1. Introduction

Cerebral palsy (CP), hereinafter referred to as “CP”, refers to a kind of nonprogressive damage that occurs during the development of fetal or infant brain tissue.^[1]^Patients often show walking dysfunction, abnormal balance ability and abnormal body stability, which is mainly caused by brain nerve damage. The recovery for children with CP mainly includes physical therapy such as exercise therapy, surgery or acupuncture and moxibustion and massage, the neurodevelopmental technique, transcranial stimulation and other recovery therapies.

Swimming, as a good exercise to develop the aerobic metabolism ability of the human body, not only promotes the heart and lung function of the body, but also promotes the improvement of unilateral muscle tone deficiency in children with CP because it requires the use of bilateral muscle strength when swimming. In addition, because of the buoyancy and resistance of the water and the pressure on the muscles from all directions when exercising in the water, it is necessary for the brain to continuously control the body to maintain balance during swimming training, which also helps to improve the balance ability of children with CP.

Since motor dysfunction is the most important clinical manifestation of children with CP, it is of great practical significance to intervene through swimming training to restore the motor ability of children with CP to a certain extent, and guide children with CP to acquire swimming skills and promote their brain function remodeling.

## 2. Case report

### 2.1. Materials and methods

#### 2.1.1. Test object.

In February 2022, 1 child diagnosed with ataxia CP by the hospital was recruited, aged 6 years and 9 months. The symptoms were: lower limb adduction and internal rotation, left neck tilt to the left due to insufficient muscle tension, and eyes squint to the right. The movement is clumsy and the coordination ability of limbs is poor; Its body balance function is poor, the sitting and standing position cannot keep the body upright and balanced for a long time, and the coordination of the random movement of hands and eyes is poor; Weak spatial cognition and orientation ability; Have persistent central motor dysfunction. When walking, the body leans forward and sideways, and the gait is staggered, which is easy to fall; In terms of expression, it shows vague language and unclear speech; Relatively retarded in intelligence.No family history of cases. The patient has received functional rehabilitation on land in the past, but the effect is not significant.

The test was approved by the Ethics Committee of Chengdu Sport University, and the families of the participants were aware of the content of the study and signed the consent form. The authors of this work have nothing to disclose. There is no diagnostic challenges and Adverse and unanticipated events.

1. Description Setup (Equipment): The first stage is mainly for swimming skill learning. Due to the peculiarities of children with CP, skill learning is carried out in a swimming pool 15 meters long and 10 meters wide; The second stage is mainly swimming training with a certain amount and intensity. The swimming pools are 25 meters long and 20 meters wide, and they are all indoor swimming pools.
2. Description location: Ande Tianyu Swimming Pool in Wuhou District, Chengdu, Sichuan Province.
3. Recruitment time: February 2022.
4. Follow-up time: the first stage of training 5 times a week, the second stage of training 5 to 6 times a week.
5. Data collection time: 1 to 2 times a week without training time for physical testing.

#### 2.1.2. Intervention program.

Routine rehabilitation training was carried out for this patient with ataxic CP, and swimming training was also taken to help the patient improve unilateral muscle tone deficiency, balance ability and body stability.

The intervention program includes 2 parts: the first stage: basic swimming skills learning; The second stage: through the implementation of swimming training with certain exercise load, the function of all parts of children with ataxic CP will be interfered (Table 1).

**Table 1:** Intervention plan at different stages.

	Water intervention	Other therapies
Phase I	(1) Perform basic balance exercises such as standing, walking, prone, supine in the water. (2) Learn the four swimming strokes.	Sensory training
Phase 2	(1) Improve each stroke technique. (2) Conduct swimming training with certain intensity and quantity. (3) Continue to carry out various resistance exercises and balance exercises in the water.	Land strength exercise: Focus on maximum strength and strength endurance; Supplemented by daily perception training

The first stage (March 1, 2022– August 31, 2022): mainly focus on learning various swimming styles, so as to carry out intensive and quantitative swimming training in the later stage. At the beginning of learning swimming stroke, it is necessary to be familiar with water exercises, such as standing in water, walking, prone, supine, changing from standing position to prone position or supine position, changing from prone position or supine position to standing position, and so on. This special environment in water is from the buoyancy of water The impact of the forces in all directions in the water on the muscles and the body and the spray generated by other swimmers when passing by lead to the obvious shaking feeling of children with CP in the water, all of which promote the exercise of balance ability of children with ataxia CP to a certain extent. The frequency of exercise is 5 classes per week, and the duration of each class is 40 minutes to 1 hour (depending on the status, learning situation and learning degree of children with ataxia CP); Exercise intensity is set to low intensity. At this stage, children mainly adapt to the water environment and learn skills.

The second stage (September 1, 2022– March 1, 2023): after the end of the first stage, that is, children with ataxic CP have learned 3 to 4 swimming strokes and have no life safety problems, enter the second stage of learning. With a certain amount of exercise intensity and amount of exercise to rehabilitate their body related functions. At this stage, the combination exercise of different swimming styles is mainly used to improve the cardiopulmonary endurance and muscle endurance of children with CP. The training method of combination exercise mainly adopts transformation training method. Through constantly changing the training load measurement, intensity and training content, to improve the abilities of all parts of children with ataxic CP; In addition, it is also supplemented with control exercises of fine movements. For example, during swimming training, children with ataxia CP are required to have finger control so that they can always pay attention to their fingers, so as to help children with ataxia CP improve their concentration.

The subject of this study is a child with ataxic CP. In addition to having certain obstacles in physical function, he also shows inattention, vague expression and mental retardation. Therefore, while taking swimming as an intervention way for physical rehabilitation, the control ability of children should be improved through the exercise of control ability. The frequency of exercise is 5 to 6 classes per week, and the duration of each class is 1 to 1.5 hours (depending on the state, learning situation and learning degree of the child with CP); The exercise intensity is set as medium intensity, and the heart rate of the child is 140 to 145 times/minutes. It is worth emphasizing that, due to the particularity of the research object, the setting of exercise intensity cannot be completely carried out according to the exercise intensity of ordinary people to improve their cardiopulmonary endurance and muscle endurance, but needs to increase the intensity step by step according to the actual situation of the children. Therefore, the heart rate of 140 to 145 beats/minutes here is based on the fact that the child has no movement basis and has certain physical defects and obstacles, and slowly increases to this intensity.

#### 2.1.3. Indicator test.

##### 2.1.3.1. Balance ability assessment

The balance ability of the human body mainly depends on the reception and processing of peripheral sensory information by the receptors of vision, proprioception and vestibular sense. The methods used in clinical balance assessment are mainly divided into 3 categories: functional test, scale assessment and balance tester. This study mainly used the Berg (Berg balance scale) balance scale to test the balance ability of this child. The test is divided into 14 items, each item is divided into 5 levels according to the degree of difficulty and is assigned 0 to 4 points.

##### 2.1.3.2. Muscle tension assessment

modified Ashworth scale was used to evaluate the left muscle tone deficiency of the child. The scores were quantified as 0, 1, 2, 3, 4, and 5. The lower the score, the lower the muscular tension of the child with CP.

##### 2.1.3.3. Walking ability assessment

1. 10-meter walking test^[2]^
  The 10-meter walking test was used to evaluate the change of walking speed before and after training. The faster the speed, the better.
2. 6-minute walk test^[3]^
  The 6-minute walking test was used to evaluate the change of walking endurance before and after training. The children walked safely at the fastest speed within 6 minutes, and the longer the walking distance, the better.

#### 2.1.4. Statistical methods.

SPSS 21.0 was used to analyze the collected data. The measurement data conforms to the normal distribution and is described with + SD; In the inter-group comparison, the paired sample *t* test was used to compare the samples before and after the intervention.

### 2.2. Intervention results

#### 2.2.1. Balance ability intervention results.

After twelve months of swimming training intervention, the training frequency is 5 days a week, 1 hour each time (but it needs to be adjusted according to the state of the subjects), and 2 days of rest. During the rest period, special imitation exercises and control exercises are still required. From the actual situation of intervention, it is effective to use the unstable environment in water to restore the balance ability of children with ataxia CP. According to the score of Berg balance scale of the child with ataxia CP, before and after the intervention, t = 5.315, *P* = .0032 (*P* < .01), indicating that the balance ability had significant differences before and after the intervention (Table 2).

**Table 2:** Breg Balace scale scoring results (mean).

Before intervention	After intervention	t	*P* value
19.55	23.97	5.315	.0032*

Paired t test.

* Indicates *P* < .05.

† Indicates *P* < .01.

#### 2.2.2. Results of unilateral muscle tension intervention.

Before the intervention, the ataxia CP child mainly showed insufficient left upper and lower limb muscle tension. The modified Ashworth scale was used to evaluate whether the unilateral muscle tension of the child with CP who participated in swimming training for twelve months was improved. Displayed from the scoring results; However, after intervention, unilateral muscle tone deficiency improved and bilateral muscle tone gradually became consistent (Table 3).

**Table 3:** Muscle tension score result table (mean).

	Before intervention	After intervention	t	*P* value
The trapezius muscle	1.87	2.03	3.01	.040*
Deltoid	2.14	2.76	4.54	.011*
Bicipital muscle of arm	1.96	2.06	3.23	.032*
Triceps muscle of arm	2.02	2.89	4.99	.008†
Latissimus dorsi	1.88	1.92	2.64	.058
Gluteus maximus	1.92	2.02	2.121	.039*
Biceps femoris	1.83	2.00	2.295	.028*
Quadriceps femoris	2.02	2.41	3.87	.018*
Gastrocnemius muscle	1.76	1.80	2.74	.052

Paired *t* test.

* Indicates *P* < .05.

† Indicates *P* < .01.

#### 2.2.3. Recovery effect of walking ability.

The results of swimming training intervention for twelve months showed that the measured data of the ataxic CP child’s physical stability were shown in Table 4 and Table 5.

**Table 4:** 10m walking test table (mean).

	Before intervention	After intervention	t	*P* value
Stride length(cm)	0.43	0.52	4.858	.001†
Step width(cm)	17.09	14.21	−5.337	.002†

Paired *t* test.

* Indicates *P* < .05.

† Indicates *P* < .01.

**Table 5:** 6 minutes walking test table (mean).

	Before intervention	After intervention	t	*P* value
Stride length(cm)	0.51	0.59	3.838	.003†
Step width(cm)	27.03	23.01	3.01	.04*

Paired *t* test.

* Indicates *P* < .05.

† Indicates *P* < .01.

## 3. Discussion

The current research on the rehabilitation treatment of children with CP mainly includes the rehabilitation treatment of their language disorders, motor disorders and activities of daily living. In the related research on the recovery of motor disorders, the recovery of balance ability, gross motor function and fine motor function is the main.

### 3.1. Relevant research results of rehabilitation effect of traditional functional training on children with CP

Functional training refers to a personalized training plan that purposefully integrates specific training skills into actions to improve the quality and ability of sports. The term functional training originated from sports medicine, which focused on rehabilitation in the early stage to improve or develop skills related to activities of daily life, and then functional training entered the field of teaching and training through sports training and applied to training facilities. Functional training is a results-oriented training, including a variety of training modes. It is defined by ACSM as the use of strength training to improve balance, coordination, strength, strength and endurance, as well as the ability to improve a person’s daily life. Michael Boyle, the famous strength and fitness coach, expressed the simplest and intuitive definition of functional training as “the most fundamental purpose is function. Therefore, functional training can be described as purposeful learning”.

Functional training is an important intervention in physical therapy. Many experts put functional training into the rehabilitation treatment plan of children with CP to improve their motor ability. Wang Hui (2004) designed a 21-week balanced and coordinated comprehensive rehabilitation training for children with ataxic CP, integrating some techniques and methods for rehabilitation treatment of children with ataxic CP. In the training, the methods of positive reinforcement, shaping, fading and token system, combined with sensory integration, and action education training, are used to complete the training objectives set at each stage. Finally, in the display of outcome indicators, the children’s body movement balance, coordination and spatial cognitive ability have been greatly improved, which proves that the effect of integrating functional training into the treatment and rehabilitation of children with CP is obvious.^[4]^The abnormal movement pattern of children with CP affects not only their movement ability, but also their balance ability. Improving balance ability and walking ability is conducive to promoting the recovery of children’s motor function. Guo Chuan (2018) randomly divided 40 patients with cerebellocerebellar stroke into routine ataxia rehabilitation training group (control group) and dynamic correction clothing combined with routine ataxia rehabilitation training group (test group). The control group adopted routine ataxia rehabilitation training, and the test group carried out routine ataxia rehabilitation training under the condition of wearing dynamic correction clothing, for a total of 6 weeks. According to the final treatment effect, both groups of patients improved the ataxia symptoms of patients with cerebellar stroke and ataxia, and the effect of the test group was more obvious, reflecting that functional training improved the balance and walking ability of the children, and improved and improved the quality of life of the patients.^[5]^Core stability plays an important role in controlling the balance ability of the human body, and core stability function training can also be used as a therapeutic means of rehabilitation training, Liang Wenrui (2018)^[6]^ In order to improve the balance ability of children with CP, the core stability training of children with CP was carried out with the help of the strap. The use of band strengthening core stability training can further improve the children’s motor ability, improve the children’s limb control function, movement balance function and muscle strength, which is consistent with the research results of Liu Can and others.^[7]^

According to many previous literature studies and the discussion of training effects, functional training has a good feedback in promoting the balance ability, stability, walking ability, muscle strength and other aspects of children with CP. It is a good rehabilitation treatment method for children with CP, promoting the recovery or improvement of motor ability of children with CP and improving their ability to live alone in daily life.

### 3.2. Results of research on the effect of swimming exercise on rehabilitation in children with CP

The application of aquatic exercise therapy in CP rehabilitation has a long history, but its development is relatively slow.^[8–10]^ Water sports therapy generally refers to the treatment of the whole body immersed in water hydrotherapy, mainly in the hydrotherapy pool, whirlpool bath, mineral bath, habad tank and swimming pool sports therapy.^[11,12]^ At present, research on the rehabilitation of children with CP using swimming (or in the water) is weak compared to the use of traditional land-based rehabilitation. Cong Ningli, et al^[13]^ (2010) A 12-year-old child with spastic CP was followed up for 4 years through swimming training. Cognitive function, language function, motor ability, self-care ability, and social adaptation were observed. Studies believe that the use of moderate to low intensity aerobic exercise is beneficial to alleviate the degree of spasticity in patients with CP; Strength training at moderate intensity improves muscle strength in the athlete’s obstacle area.^[14]^ Wang Guoxiang, et al^[15]^ (2017) After hydrotherapy in a 10-year-old child with spastic CP, it was found that the gross motor function and balance of the child were improved, and the main mechanism was the stimulation of buoyancy, water purification pressure and water vortex impact.

Studies have shown that swimming training or other aquatic therapies have a certain healing effect on children with CP. However, at present, the rehabilitation treatment of swimming for children with CP is still mainly adolescents and mainly focuses on children with spastic CP, and the rehabilitation therapy of children with other types of CP is less, and the intervention program of swimming training for children with other types of CP is weaker. However, from previous research results, it has been found that the earlier the rehabilitation intervention for children with CP, the better, and the recovery function effect in all aspects may be better. In addition, rehabilitation interventions for children with CP should be based on the exploration of their neural mechanisms. Based on the above induction and summary, this study studies the swimming training intervention treatment of a child with ataxia CP aged 6 years and 9 months, so as to enrich the rehabilitation treatment plan of children with CP.

### 3.3. Neurological mechanism of swimming exercise intervention on recovery effect in children with CP

In recent years, sports training has made important progress in the rehabilitation treatment of children with CP. According to the hypothesis of brain plasticity, the hypothesis of action experience and the explanation of multi-channel theory,^[16,17]^ it is found that exercise training can promote the recovery of neural function in children with CP, and its mechanism is mainly related to neural plasticity. The main manifestations are as follows: it causes hemodynamic changes, thus improving the blood supply function of the brain^[18,19]^; Promote the intellectual development of mentally retarded children^[20]^; It increases the production of neurotrophic factors in the brain, thus promoting the repair of the ultrastructure of the nerve synapse, and thus promoting the regeneration or remodeling of nerve cells_._^[21–23]^

In previous animal experiments, it has been found that exercise can change the neurotransmitters in functional brain areas such as the forebrain, striatum and hippocampus, thus improving the cognitive ability of the brain.^[24]^ Swimming can promote the neural protection and plasticity mediated by microtubule dynamics in mice with intracerebral hemorrhage.^[25]^ Yao Hongen, et al^[26]^ (2005) found that regular swimming is an appropriate exercise method that can improve the functional state of the whole body and increase the functional activity of the central nervous system after 2 weeks of basic swimming training for 9 rats. The mechanism of regular swimming to promote the establishment of animal operant conditioning may be achieved by improving the glutamate transmitter and GABA transmitter in the central nervous system.

In human experiments, scholars used fNIRS to detect the sensitive changes of prefrontal cortex activation in children with unilateral CP after hand training, indicating that motor function was improved^[27]^; Other scholars (Friedl-Werner et al^[28]^, 2020) used FMRI technology to speculate about the dysfunction caused by lack of physical activity.

However, at present, the relevant research on swimming training intervention in motor function rehabilitation of children with CP is relatively weak, and the discussion on its neural mechanism has not been thoroughly clarified. The main research object of intervention by swimming is animal experiment. There are few experiments of direct intervention on human body, and a few experiments of rehabilitation on human body have not explained the specific recovery objectives and implementation of intervention programs. Therefore, this study intervened in the recovery of muscle tension, balance ability and walking ability of children with CP through swimming training.

## 4. Result

In previous studies, it has been found that glutamate is the transmitter of most synapses in the central nervous system. Then swimming proved its important role in the establishment of conditioned reflex. The mechanism of the establishment of conditioned reflex is mainly the change of the basic concentration of neurotransmitter, which promotes the establishment and formation of conditioned reflex. Therefore, when swimming training is used to rehabilitate some functions of children with ataxic CP, it is necessary to increase the basic concentration of neurotransmitters through aerobic metabolism exercise according to this rule to promote the memory ability of children with ataxic CP in brain areas, and then improve the cognitive function of children with ataxic CP.

### 4.1. Balance ability recovery effect

After the first stage of practice in the environment of unstable support in the water, this study improved the balance ability of the child with CP through continuous practice of standing, walking, prone, supine, and from standing to prone or supine, from prone or supine to standing. The test of balance ability mainly adopts the Breg balance scale balance scale. The results showed that the balance ability of the child with ataxic CP was improved to a certain extent by constantly changing his body posture in the water, constantly creating an unbalanced environment in the water, such as the effects of waves, spray, forces in different directions, and randomly switching various body postures in the water to maintain balance.

### 4.2. Recovery effect of unilateral muscular dystonia

The main symptoms of insufficient muscle tension are that the muscles cannot exert the strength they should have and how long they will last after exerting the strength. Therefore, the moderate load intensity endurance training is adopted for its recovery training. The benefits are: First, endurance training includes cardiopulmonary endurance and muscle endurance. After a long and targeted endurance training, it will help to solve the problems of the children’s muscle endurance and cardiopulmonary endurance, and then solve the problem of their unilateral muscle tension deficiency, which cannot maintain a certain posture or do a certain action for a long time; Second, it is conducive to the growth of its strength. At the same time, the load intensity should not be too large because of the physical obstacle or defect of the child. Proper load can help improve the muscle endurance and maximum strength of children with ataxic CP, and then solve the problems of insufficient balance ability and trunk stability caused by unilateral muscle tension deficiency. Thirdly, due to the hardship of endurance training itself, it is helpful to improve the will quality of children with CP and overcome the discomfort caused by the external environment.

### 4.3. Recovery effect of walking ability

After 6 months of swimming training, the physical stability of the child with CP was improved to some extent. The main performance is: due to the improvement of muscle endurance, the duration of unilateral muscle tension maintaining a certain posture or doing a certain action increase, which makes the time for the trunk to maintain a stable posture longer. At the same time, after the problem of insufficient left muscle tone was improved, the head no longer tilted to the left, and then the eyes gradually got used to looking straight ahead, which solved the problem of strabismus to a certain extent. The improvement of muscle endurance, the improvement of left muscle tension deficiency and the correction of strabismus made the trunk stability of the child with CP improved.

In the early stage of intervention, because the child did not carry out intensive training, he was unwilling to cooperate in the early stage of training, always crying, and was more sensitive. However, as the child gradually adapts to this training mode in the later stage, the child can complete the training according to the instructions.

And after completing the training, there was no discomfort.

After intervention, it was found that the child with CP had significantly improved his balance, gait stability and unilateral muscle tone insufficiency, and his daily walking could maintain basic balance, basically achieve the condition of not falling, and be able to complete daily activities independently.

## 5. Conclusion and prospects

### 5.1. Research conclusion

In this paper, swimming training is used as an intervention to rehabilitate some functions of children with ataxia CP. The study found that swimming has a significant effect on the rehabilitation of children with ataxia CP. The specific performance is as follows: The water environment is relatively special, and the unstable factor of the force from different directions in the water can be used to rehabilitate the balance ability of children with ataxia CP; Strengthened muscle endurance training has obvious recovery effect on children with CP who has insufficient unilateral muscle tension, and after a certain degree of recovery, the bilateral muscle tension gradually tends to be consistent, which is more conducive to the improvement of trunk stability and body posture balance ability of children with CP; Use moderate load intensity for endurance training. After learning the basic swimming posture and having no life safety problems, maintaining the training intensity at the heart rate of 140 to 145 beats/minutes has a significant effect of improving the cardiopulmonary endurance and muscle endurance of children with ataxia CP; Taking swimming training as an intervention to rehabilitate children with ataxia CP can not only help the recovery of some body functions of the children to a certain extent, but also exercise the children’s will quality and the ability to overcome the discomfort of the external environment, so as to help the children adapt to the difficulties in real life.

The family members affirmed the effectiveness of the intervention in this study and found that the child with CP had significant improvements in balance ability, gait stability, and unilateral muscle tone deficiency. They decided to continue swimming training.

### 5.2. Research deficiency and prospect

There are also some shortcomings in this study. The specific manifestations are as follows: This study is only a case study, reporting only 1 child with ataxia CP, so there are certain limitations in the sample size. This study will continue to conduct longitudinal follow-up to observe the subsequent development of the subjects physical function and physical fitness, providing more reference for the rehabilitation of children with CP in this type.

The research mainly focuses on children with ataxia type CP, and further research needs to be extended to other types of CP children to observe the intervention effect of swimming training on different types of CP children, in order to further promote the universality of the method.

## Author contributions

**Conceptualization:** Shuang Hao.

**Supervision:** Shuang Hao.

**Software:** Yuxuan Wang.

**Writing – original draft:** Jing Zeng.

**Writing – review & editing:** Qing Liu.

## References

[1] Wang J Yue L Chen ZH. Study on the efficacy of suspension training combined with MOTOmed intelligent training in the rehabilitation of children with spastic cerebral palsy. Chin J Child Health. 2022;30:240–243 + 263.

[2] Cho C Hwang W Hwang S. Treadmill training with virtual reality improves gait, balance, and muscle strength in children with cerebral palsy. Tohoku J Exp Med. 2016;238:213–8. 26947315 10.1620/tjem.238.213

[3] Mylius CF Paap D Takken T. Reference value for the 6-minute walk test in children and adolescents: a systematic review. Expert Rev Respir Med. 2016;10:1335–52. 27817221 10.1080/17476348.2016.1258305

[4] Wang H. A case study on rehabilitation training for children with cerebral palsy-balance and coordination training for children with ataxia. Spec Educ China. 2004;49:83–7.

[5] Guo C Wang J Li XZ. Effects of dynamic orthodontics combined with conventional rehabilitation training on balance and walking ability in patients with cerebellar stroke and ataxia. Chin J Rehabil Med. 2018;33:1290–4.

[6] Liang WR Wu XP Zhang QF. Effect of band strengthening core stability training on motor function and balance in children with spastic cerebral palsy. Chin Rehabil Theory Pract. 2018;24:97–100.

[7] Liu C Wang PQ Qing R. Application of core control training in pediatric cerebral palsy. Chin Rehabil Theory Pract. 2013;19:881–2.

[8] Hutzler Y Chacham A Bergman U. Effects of a move-ment and swimming program on water orientation skills and self-concept of kindergarten children with cerebral palsy. Percept Mot Skills. 1998;86:111–8. 9530718 10.2466/pms.1998.86.1.111

[9] Hutzler Y Chacham A Bergman U. Effects of a move-ment and swimming program on vital capacity and water orien-tation skills of children with cerebral palsy. Dev Med Child Neurol. 1998;40:176–81. 9566654 10.1111/j.1469-8749.1998.tb15443.x

[10] Song FX Li XJ Cheng CF. Effects of hydrotherapy on gross motor function and lower limb muscle strength and muscle tone in children with spastic diplegia and cerebral palsy. Pediatr Integr Tradit Chin West Med China. 2015;7:331–3.

[11] Fortune N Madden R Almborg AH. Use of a new interna-tional classification of health interventions for capturing infor-mation on health interventions relevant to people with disabili-ties. Int J Environ Res Public Health. 2018;15:145. 29342077 10.3390/ijerph15010145 PMC5800244

[12] World Health Organization. International Classification of Health Interventions (ICHI) [EB/OL]. 2020. Available at: https://mitel.dimi.uniud.it/ichi/#http://id.who.int/ichi/entity/619012253.

[13] Cong F Cui Y. Clinical practice guidelines for aquatic exercise therapy in children with cerebral palsy. Chin Rehabil Theory Pract. 2021;27:1–14.

[14] Cong NL Guan ZX. Application research on the effect of swimming training on comprehensive rehabilitation of cerebral palsy. J Chengdu Inst Phys Educ. 2010;36:58–62.

[15] Wang GX Liang B Tao R. Motor function assessment and hydrotherapy scheme for children with cerebral palsy based on ICF-CY. Chin Rehabil Theory Pract. 2017;23:146–50.

[16] Chen AG Yan J Yin HC. Development and Practice of Physical Activity Program to Improve Children’s Executive Function. Changsha: Hunan People’s Publishing House; 2015:39–54.

[17] McMorris T Sproule J Turner A. Acute, intermedi- ate intensity exercise, and speed and accuracy in working memory tasks: a meta-analytical comparison of effects. Physiol Behav. 2011;102:421–8. 21163278 10.1016/j.physbeh.2010.12.007

[18] Zhang P Yu H Zhou N. Early exercise im- proves cerebral blood flow through increased angiogene-sis in experimental stroke rat model. J Neuroeng Rehabil. 2013;10:43. 23622352 10.1186/1743-0003-10-43 PMC3648391

[19] Hu X Zheng H Yan T. Physical exercise in-duces expression of CD31 and facilitates neural function recovery in rats with focal cerebral infarction. Neurol Res. 2010;32:397–402. 20483007 10.1179/016164110X12670144526309

[20] Polesia GV. Peculiarities of the electrical activity of the brain in schizophrenic patients following the pursuit of therapeutic gymnastics. Zh Nevropatol Psikhiatr Im S S Korsakova. 1966;66:928–32. 6000194

[21] Zhang L Hu X Luo J. Physical exercise im-proves functional recovery through mitigation of autophagy, attenuation of apoptosis and enhancement of neurogenesis after MCAO in rats. BMC Neurosci. 2013;14:46. 23565939 10.1186/1471-2202-14-46 PMC3637142

[22] Zheng HQ Zhang LY Luo J. Physical exercise promotes recovery of neurological function after ischemic stroke in rats. Int J Mol Sci. 2014;15:10974–88. 24945308 10.3390/ijms150610974 PMC4100192

[23] Pan X Jiang T Zhang L. Physical exercise pro-motes novel object recognition memory in spontaneously hypertensive rats after ischemic stroke by promoting neural plasticity in the entorhinal cortex. Front Be-hav Neurosci. 2017;11:185. 10.3389/fnbeh.2017.00185 PMC5682296 29167635

[24] Ohkuwa T Sato Y Naoi M. Glutathione status and reactive oxygen generation in tissues of young and old exercised rats. Acta Physiol Scand. 1997;159:237–44. 9079154 10.1046/j.1365-201X.1997.576351000.x

[25] Sun LM Wang GH Wu ZT. Swimming exercise reduces the vulnerability to stress and contributes to the AKT/GSK 3β/CRMP 2 pathway and microtubule dynamics mediated protective effects on neuroplasticity in male C57BL/6 mice. Pharmacol Biochem Behav. 2021;211:173285. 34626621 10.1016/j.pbb.2021.173285

[26] Yao HE Liu K Rong XJ. Study on the neural mechanism of regular swimming promoting the establishment of operational conditioning in rats. J Tianjin Inst Phys Educ. 2005;20:13–7.

[27] Surkar SM Hoffman RM Willett S. Hand-arm bimanual intensive therapy improves prefrontal cortex activation in children with hemiplegic cerebral palsy. Pediatr Phys Ther. 2018;30:93–100. 29578992 10.1097/PEP.0000000000000486

[28] Friedl-Werner A Brauns K Gunga H-C. Exercise-induced changes in brain activity during memory encoding and retrieval after long-term bed rest. Neuroimage. 2020;223:117359. 32919056 10.1016/j.neuroimage.2020.117359

